# Lack of Postprandial Peak in Brain-Derived Neurotrophic Factor in Adults with Prader-Willi Syndrome

**DOI:** 10.1371/journal.pone.0163468

**Published:** 2016-09-29

**Authors:** Marta Bueno, Susanna Esteba-Castillo, Ramon Novell, Olga Giménez-Palop, Ramon Coronas, Elisabeth Gabau, Raquel Corripio, Neus Baena, Marina Viñas-Jornet, Míriam Guitart, David Torrents-Rodas, Joan Deus, Jesús Pujol, Mercedes Rigla, Assumpta Caixàs

**Affiliations:** 1 Department of Endocrinology and Nutrition, Hospital Universitari Arnau de Vilanova, Lleida, Spain; 2 Specialized Service in Mental Health and Intellectual Disability, Institut Assistència Sanitària (IAS), Parc Hospitalari Martí i Julià, Girona, Spain; 3 Department of Endocrinology and Nutrition, Sabadell University Hospital, Corporació Sanitària Parc Taulí, Sabadell, Spain, Autonomous University of Barcelona, Bellaterra, Spain; 4 Mental Health Center, Corporació Sanitària Parc Taulí, Sabadell, Spain; 5 Pediatric Endocrinology Unit, Sabadell University Hospital, Corporació Sanitària Parc Taulí, Sabadell, Spain; 6 Genetics Laboratory, UDIAT, Corporació Sanitària Parc Taulí, Sabadell, Spain; 7 Department of Clinical and Health Psychology, Autonomous University of Barcelona, Bellaterra, Spain; 8 MRI Research Unit, Department of Radiology, CIBERSAM G21, Hospital del Mar, Barcelona, Spain; 9 Guttmann Neurorehabilitation Institute, Barcelona, Spain, Autonomous University of Barcelona, Bellaterra, Spain; National Brain Research Centre, INDIA

## Abstract

**Context:**

Prader-Willi syndrome (PWS) is characterized by severe hyperphagia. Brain-derived neurotrophic factor (BDNF) and leptin are reciprocally involved in energy homeostasis.

**Objectives:**

To analyze the role of BDNF and leptin in satiety in genetic subtypes of PWS.

**Design:**

Experimental study.

**Setting:**

University hospital.

**Subjects:**

90 adults: 30 PWS patients; 30 age-sex-BMI-matched obese controls; and 30 age-sex-matched lean controls.

**Interventions:**

Subjects ingested a liquid meal after fasting ≥10 hours.

**Main Outcome Measures:**

Leptin and BDNF levels in plasma extracted before ingestion and 30’, 60’, and 120’ after ingestion. Hunger, measured on a 100-point visual analogue scale before ingestion and 60’ and 120’ after ingestion.

**Results:**

Fasting BDNF levels were lower in PWS than in controls (p = 0.05). Postprandially, PWS patients showed only a truncated early peak in BDNF, and their BDNF levels at 60' and 120' were lower compared with lean controls (p<0.05). Leptin was higher in PWS patients than in controls at all time points (p<0.001). PWS patients were hungrier than controls before and after eating. The probability of being hungry was associated with baseline BDNF levels: every 50-unit increment in BDNF decreased the odds of being hungry by 22% (OR: 0.78, 95%CI: 0.65–0.94). In uniparental disomy, the odds of being hungry decreased by 66% (OR: 0.34, 90%CI: 0.13–0.9). Postprandial leptin patterns did no differ among genetic subtypes.

**Conclusions:**

Low baseline BDNF levels and lack of postprandial peak may contribute to persistent hunger after meals. Uniparental disomy is the genetic subtype of PWS least affected by these factors.

## Introduction

Prader-Willi syndrome (PWS) is a genetic disorder caused by the lack of expression of the paternally inherited genetic material located at 15q11-q13. This lack of expression is due to a deletion (subtype I or II) of a region of the paternally inherited chromosome 15 in 65% to 75% of cases, to maternal uniparental disomy (UPD) in 20% to 30% (both copies of chromosome 15 are inherited from the mother), or to an imprinting defect in 1% to 3% [1]. The clinical features include neonatal hypotonia; intellectual disability; challenging behavior in response to changes in routine; psychiatric features [2]; endocrine disorders, such as growth hormone deficiency or hypogonadotropic hypogonadism; and the absence of satiety, which leads to early childhood obesity [3] Behaviorally, individuals with deletion exhibit more severe problems than those with UPD, including skin-picking and higher obsession for food [4]; by contrast, individuals with UPD display higher levels of psychosis and social impairments [4].

Many studies aiming to elucidate the potential causes of hyperphagia in PWS have identified factors that might contribute to the absence of satiety in PWS, including high levels of the orexigenic hormone ghrelin persisting in the postprandial state, low levels of pancreatic polypeptide, and a lack of the postprandial polypeptide YY peak [5–11]. Leptin, a hormone secreted by adipose cells, regulates appetite by stimulating anorexigenic neurons and inhibiting orexigenic neurons in the arcuate nucleus of the hypothalamus. Obese individuals with or without PWS have high leptin levels and leptin resistance [12–15]. However, some inconsistencies exist across studies [16,17], and the role of these peptides remains unclear [17]. Although other molecules that could explain the lack of satiety in PWS have been sought, to date, our understanding of this problem remains poor due to the complexity of the hunger-satiety circuitry ([8–20].

Brain-derived neurotrophic factor (BDNF) is a neurotrophin involved in the development and plasticity of the central nervous system [21]. BDNF is stored in platelets, from where it is released into the peripheral circulation [22]. Leptin and BDNF play reciprocal roles in inducing satiety: leptin regulates BDNF expression, and BDNF plays a role in conveying the leptin anorexigenic signal by regulating the formation or function of neuronal connections in brain centers controlling energy balance [23]. BDNF is found in key brain regions involved in eating behavior, energy homeostasis, and weight control [24], and it has been linked to obesity and insulin resistance [25].

Animal models have demonstrated that BDNF haploinsufficiency leads to a reduction in hypothalamic expression of BDNF mRNA that causes hyperphagia and obesity that could be reversed by intracerebroventricular infusions of BDNF [26]. In humans, the loss of one copy of the *BDNF* gene, caused by deletion of the region containing the gene in the WAGR (Wilms tumor, aniridia, genitourinary anomalies, and intellectual disability) syndrome or by a chromosomal inversion, is associated with syndromic phenotypes linked to hyperphagia and obesity [27,28]. Moreover, in humans, the *BDNF* p.Val66Met single nucleotide polymorphism has been associated with obsessive-compulsive disorders [29], bipolar affective disorder [30], and eating disorders such as anorexia nervosa or bulimia nervosa [31]. One study found obese children had lower fasting BDNF than lean controls, and BDNF increased after lifestyle intervention [32]. Another study found children with PWS had lower fasting BDNF than obese controls [33]. To our knowledge, BDNF has not been studied in adult patients with PWS, and postprandial BDNF levels have not been studied in obese patients with or without PWS.

Thus, we aimed to evaluate whether low BDNF levels could contribute to the pathogenesis of hyperphagia and obesity in PWS. To this end, we measured fasting and postprandial BDNF and leptin concentrations in plasma in adults with different genetic subtypes of PWS and compared them with matched obese and healthy controls.

## Subjects and Methods

### Subjects

We studied 30 adults with PWS, 30 obese controls matched for age, sex, and BMI, and 30 lean subjects matched for age and sex. All subjects were Caucasian and their weight was stable for at least 3 months before inclusion in the study. PWS patients and obese controls were recruited from the Endocrinology and Nutrition Department, and lean subjects were hospital staff or acquaintances that participated voluntarily. All subjects or their parents/guardians (in PWS patients) provided written informed consent. The Institutional Ethics Committee of Corporació Sanitària Parc Taulí approved the protocol, and all investigations complied with the Helsinki Declaration.

Five men with PWS were undergoing testosterone replacement for hypogonadism. Five women with PWS were undergoing estrogen and progestagen therapy for hypogonadism; all these were studied in the follicular phase. Although 14 subjects with PWS had been treated with growth hormone until puberty, none were receiving growth hormone at the time of the study. Seven subjects with PWS and one obese control had type 2 diabetes mellitus.

Intellectual disability in PWS subjects was mild in 77.4% and moderate in 22.6%.

### Experimental methods

Before the study, PWS patients’ genetic diagnosis was established by methylation specific PCR (MS-PCR) confirming the absence of the paternal allele at 15q11-q13, and deletion status was established by fluorescence in situ hybridization. Deletions were classified as subtype (I or II) using methylation-sensitive multiplex ligation-dependent probe amplification (MS-MLPA; MRC-Holland; Amsterdam, The Netherlands). Microdeletions of the imprinting center were also identified. In the absence of deletions, we analyzed multiple microsatellite markers distributed inside the 15q11-q13 region and along chromosome 15 in the probands and their parents to distinguish maternal UPD. When biparental inheritance was found, UPD was ruled out, and PWS was attributed to an epigenetic imprinting defect.

For the baseline and postprandial study, subjects were admitted to the Unit at 8 a.m. after an overnight fast of at least 10 hours. Anthropometrical parameters were determined by bioelectrical impedance analysis (TANITA, body composition analyzer BC-418 MA Biologica Tecnologia Medica SL-BCN).

A catheter was inserted into the antecubital vein, and the line was maintained with saline infusion. Blood samples were drawn for the fasting study. For the postprandial study, subjects ingested a standard liquid meal (Resource 2.0, Nestle Lab, 1200 kcal, 43% carbohydrate, 39% fat, 18% protein) and blood samples were drawn at 0, 30, 60 and 120 min. All samples were collected on ice and spun at 4°C. Plasma was centrifuged and stored at 80°C until processed.

Assays were performed using commercially available methods. BDNF was quantified by an enzyme-linked immunosorbent assay (ELISA) kit (Cat. No. CYT306, Millipore; Billerica, MA, USA) following the manufacturers’ instructions. This kit’s intra-assay variation is ±3.7% and its inter-assay variation is ±8.5% (125pg/mL). Leptin was determined using Luminex 200 and the Human Metabolic Hormone Magnetic Bead Panel (HMHMAG-34K, Merck Millipore; Billerica, MA, USA) following the manufacturer’s instructions. This kit’s intra-assay variation is ±7% and its inter-assay variation is ±10% (184 pg/mL).

Before the meal and 60’ and 120’ after the meal, subjects quantified their hunger on a visual analogue scale ranging from 0 to 100.

### Statistical methods

To compare groups, we used ANOVA test for normally distributed variables and the Kruskal-Wallis test for non-normally distributed variables.

We elaborated a general linear regression model with repeated measures for each peptide at baseline that included the following covariates: sex, BMI, percentage of body fat, glucose, and insulin.

We elaborated a logistic model for the binary indicator of being hungry after the meal. Results are reported as odds ratios (OR) with corresponding 95% confidence intervals.

We used SAS system v9.2 (SAS Institute Inc., Cary, NC, USA) for all analyses.

## Results

### Fasting study

Table 1 reports subjects’ baseline characteristics. Lean subjects had lower values for BMI, waist circumference, and percentage of body fat than the subjects in the other two groups.

**Table 1:** Baseline characteristics of all groups.

	PWS (n = 30)	Obese controls (n = 30)	Lean controls (n = 30)
**Sex (M/F)**	15/15	15/15	15/15
**Age (yr)**	27.5±8.02	28.4±7.13	27.9±7.77
**BMI (kg/m**^**2**^**)**	32.4±8.14	33.7±6.88	22.1±2.05*^$^
**Body fat (%)**	37.0±8.39	35.7±9.70	20.3±7.23*^$^
**Waist (cm)**	105.0±18.37	105.3±15.14	78.3±7.43*^$^
**Glucose (mg/dL)**	94.1±22.03	98.20±32.08	86.7±6.64*^$^
**Insulin (μU/mL)**	333.7±218.5	493.19±213.7	304.8±134.7
**HOMA-IR Index**	1.69±0.84	2.94±1,57	1.69±0.77
**Triglyceride (mg/dL)**	89 (64–134)	83 (66–108)	61 (48–69)*
**Platelets (x10**^**9**^**/L)**	256.2±69.0	256.9±61.3	230.2±46.4

p <0.05 vs

* PWS patients

^$^ obese patients.

All quantitative values are expressed as means±SD except for triglycerides, expressed as medians (interquartile range).

PWS: Prader-Willi Syndrome; BMI: body mass index; HOMA-IR: homeostatic model assessment insulin resistance.

Fasting glucose levels were lower in lean subjects than in PWS patients and obese controls. There were no significant differences among the three groups in fasting insulin or insulin-resistance determined by homeostatic model assessment (HOMA-IR-index). Fasting triglycerides were higher in PWS subjects than in lean controls, although they were within the normal range in all subjects (Table 1).

**Fig 1:**
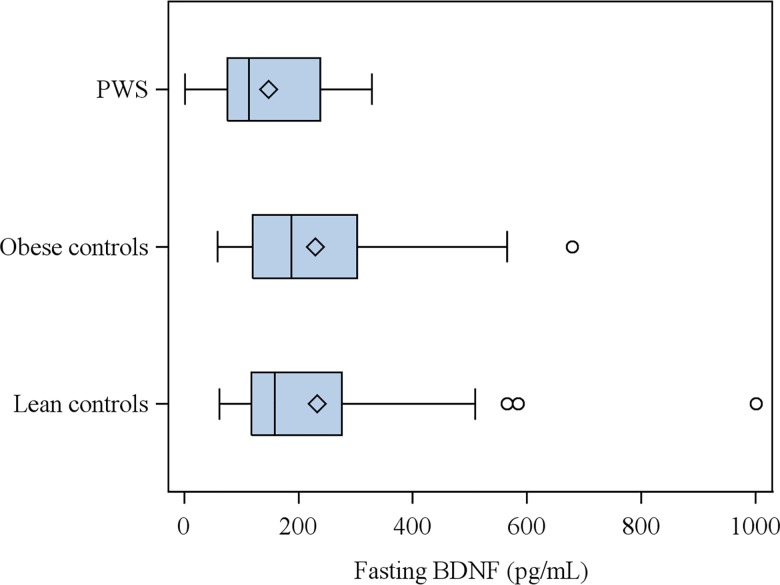
Plasma Brain-derived neurotrophic factor (BDNF) levels in subjects with Prader-Willi syndrome (PWS) and in obese and lean control subjects. PWS subjects vs. obese and lean controls, P = 0.05.

Plasma BDNF levels at fasting were lower in PWS patients than in obese and lean controls (Χ^2^ = 5.785 p = 0.05). In fact, median BDNF for PWS was 113.7 pg/mL, more than 30% lower than the median for the control groups (obese: 187.6 and lean: 158.2 pg/mL). Moreover, the distribution of BDNF values in PWS subjects was more homogeneous and symmetric than in the other two groups (Fig 1). Correcting BDNF concentrations by the number of platelets did not modify the results (data not shown).

**Fig 2:**
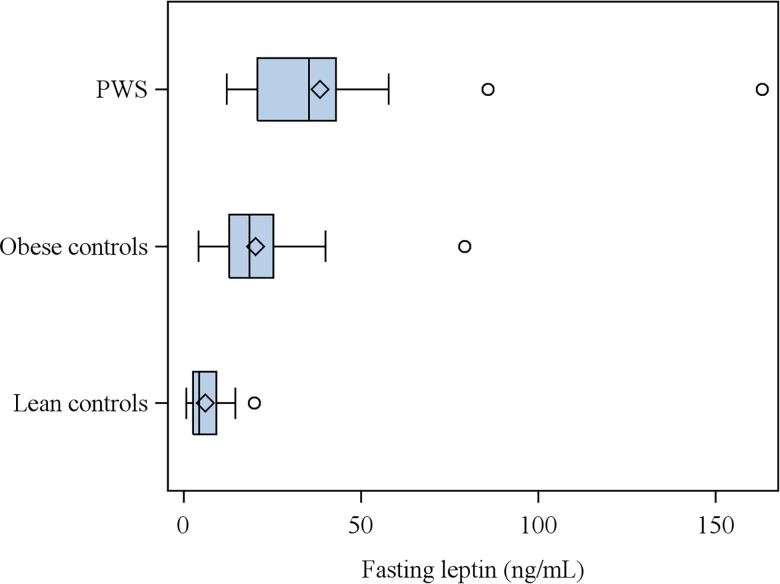
Plasma BDNF levels in subjects with Prader-Willi syndrome (PWS) and in obese and lean control subjects. P<0.01 PWS subjects vs. obese and lean controls, obese controls vs. lean controls.

In the fasting study, leptin distribution was asymmetric and PWS subjects showed more variability than obese and lean controls (Fig 2). The median of leptin was higher in PWS patients than in the other groups (PWS: 35.3, obese: 18.5, and lean 4.41 ng/mL; Χ^2^ = 55.72, p<0.001).

As expected, in the baseline model for leptin controlling for sex, BMI, percentage of body fat, glucose, and insulin, we found a significant effect for insulin (an increase of 100 μU/mL in insulin increased leptin by 2 ng/mL; p = 0.033).

To evaluate the relationship between BDNF and leptin levels, we analyzed the BDNF/leptin quotient, which is a relative measure of BDNF levels per unit of leptin. The BDNF/leptin quotient was lowest in PWS patients (PWS: 3.9 (1.6–8.8) vs Obese: 11.1 (5.5–25.9) vs Lean: 35.3 (19.6–76.8); Χ^2^ = 44.79, p<0.001).

### Postprandial study

Glucose, insulin, and triglyceride increased postprandially in all groups (Data not shown).

**Fig 3:**
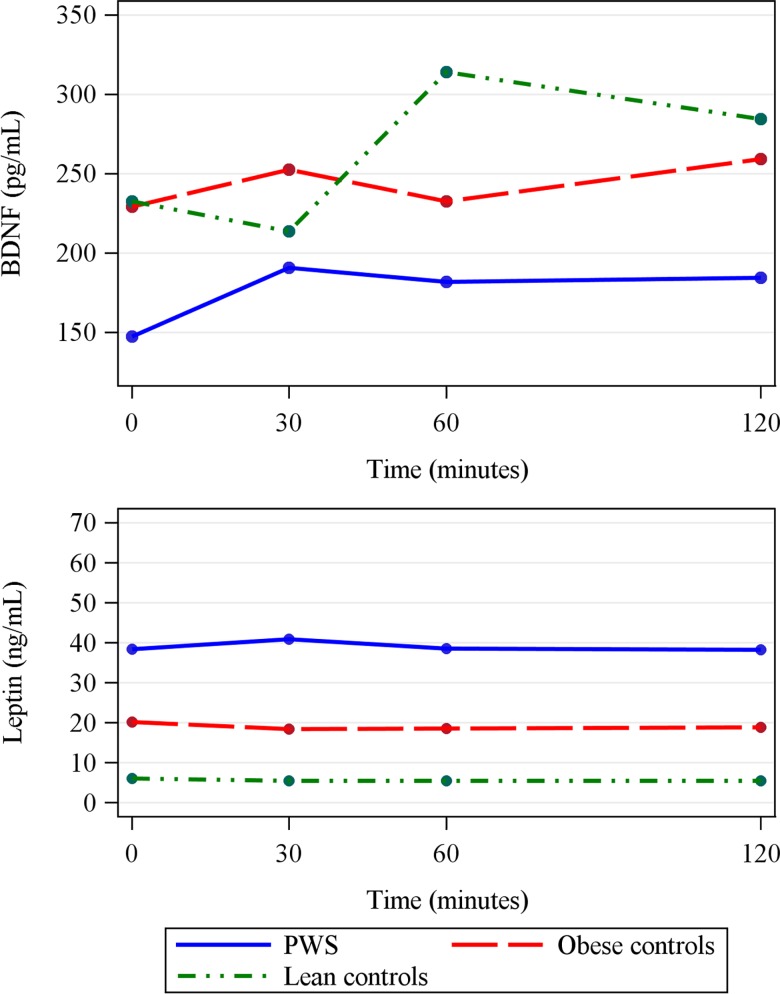
**Postprandial BDNF (Brain-derived neurotrophic factor) (A) and leptin (B) levels in subjects with Prader-Willi syndrome (PWS), in obese controls, and in lean controls.** A peak in BDNF at 60 min was observed only in lean subjects (p<0.05 baseline vs 60 min in lean controls). Subjects with PWS had lower BDNF levels at 60 and 120 min than lean controls (p<0.05). Leptin levels within groups did not change between baseline and postprandial measurements. PWS subjects had higher leptin levels than the other groups at all time points (p<0.001).

In lean subjects, postprandial BDNF peaked 60' after the liquid meal (p<0.05). However, in subjects with PWS, there was only a truncated early BDNF peak, and BDNF levels were lower than in lean controls 60' and 120' after the meal (estimatediff_60_ = 132.24; estimatediff_120_ = 99.94, p<0.05). In obese controls, postprandial BDNF pattern was intermediate between PWS and lean controls, that is, similar to PWS patients with the early truncated peak, but without significant differences at any time after the meal when compared to lean controls (Fig 3A).

Subjects with PWS had higher leptin levels than obese and lean controls during the postprandial study (estimatediff from 12.97–35.37, p<0.05 at all time points), without significant changes in leptin concentrations over time in any groups (Fig 3B).

### Relationship between hunger and levels of BDNF and leptin

Both before and after the meal, PWS subjects were hungrier than subjects in the other groups. Hunger was scored from 0 to 100 as mentioned in the methods section. (Fasting: PWS: 68.5±26.2, obese: 45.7±26.5, lean: 58.8±20.3, p<0.05; 60’ postprandial: PWS: 36±37.3, obese: 8.3±11.4, lean: 6.5±12.9, p<0.05, 120’ postprandial: PWS: 43.7±37.9, obese: 9.83±17.1, lean: 6.53±13.9, p<0.05).

**Fig 4:**
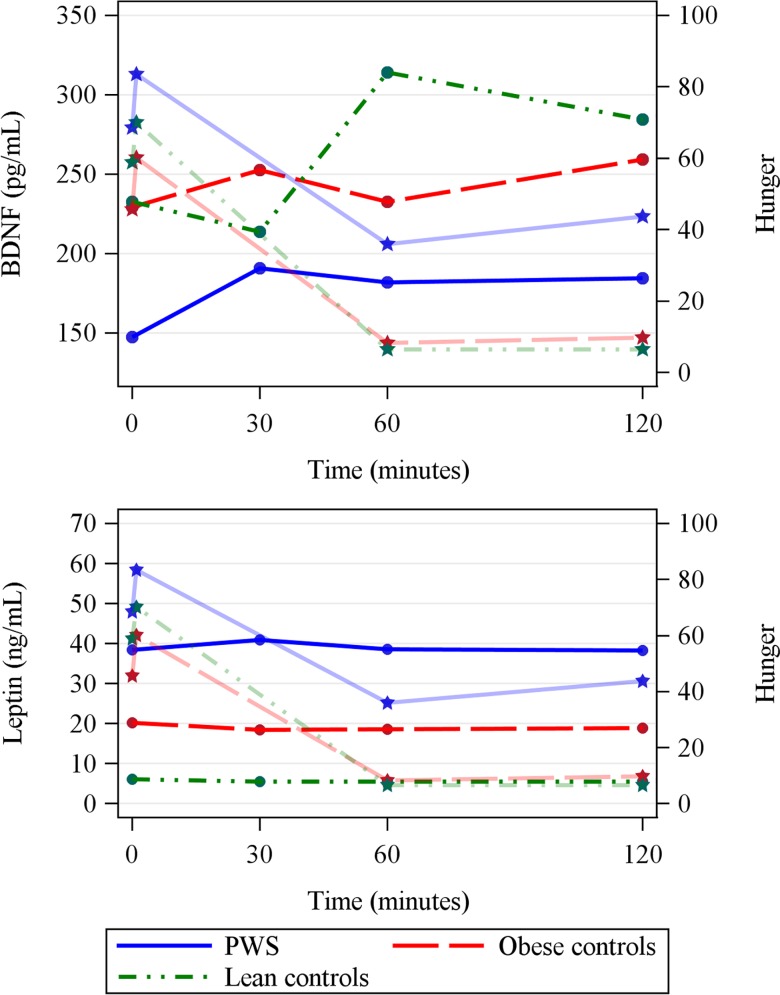
**Fasting and postprandial plasma Brain-derived neurotrophic factor (BDNF) (A) and leptin (B) levels (circles) in the three groups, overlaid with hunger score (stars) over time.** Note that postprandial BDNF peak in lean controls coincides with the lowest hunger score. PWS = Prader Willi Syndrome. Hunger was quantified on a visual analogue scale ranging from 0 to 100.

In lean subjects, the postprandial BDNF peak at 60’ coincided with the lowest hunger score. However, in PWS patients and in obese controls, the truncated early BDNF peak at 30’ did not coincide with the lowest hunger score observed at 60’ (Fig 4A); moreover, postprandial leptin levels over time were not associated with hunger scores in neither group (Fig 4B).

The logistic regression model found an association between postprandial hunger and baseline BDNF levels in PWS subjects: for every 50 units increment in BDNF, the odds of being hungry decreased by 22% (OR: 0.78, 95% CI: 0.65–0.94) and for every 10 units increase in baseline BDNF/leptin quotient, the odds of being hungry decreased by 55% (OR: 0.44, 95% CI: 0.23–0.84)

### Genetic subtype and BDNF, leptin, and hunger

The genetic tests confirmed 20 paternal deletions (7 subtype I and 13 subtype II), 7 UPDs, and 3 imprinting defects.

**Fig 5:**
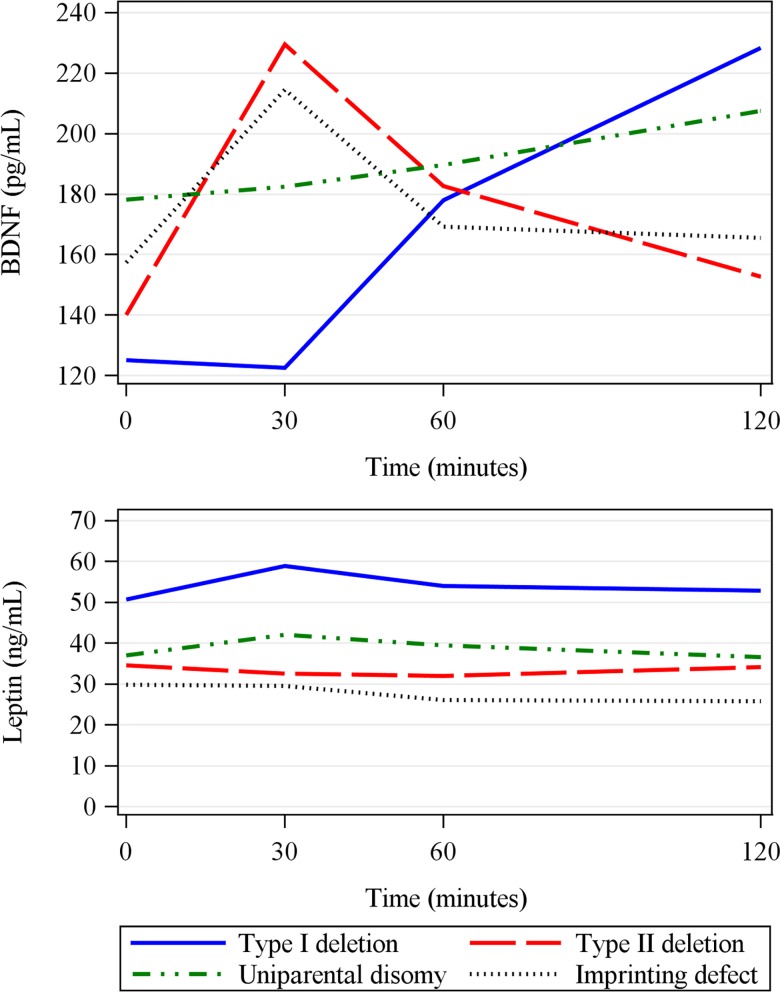
**Baseline and postprandial Brain-derived neurotrophic factor (BDNF) (A) and leptin (B) levels in plasma by genetic subtype.** Note that uniparental disomy is the only genetic subtype with sustained BDNF levels.

In the fasting state, BDNF levels were similar in all genetic subtypes. However, in the postprandial state, we observed small differences among subtypes in the pattern of BDNF levels over time (Fig 5A). Subjects with type II deletion or imprinting defects had an early truncated BDNF peak 30 minutes after the meal, followed by a decrease to baseline levels. However, subjects with type I deletion showed a delayed increase in BDNF levels. In subjects with UPD, BDNF levels remained unchanged over time with a tendency to rise. No differences in postprandial leptin patterns were observed among genetic subtypes (Fig 5B).

**Fig 6:**
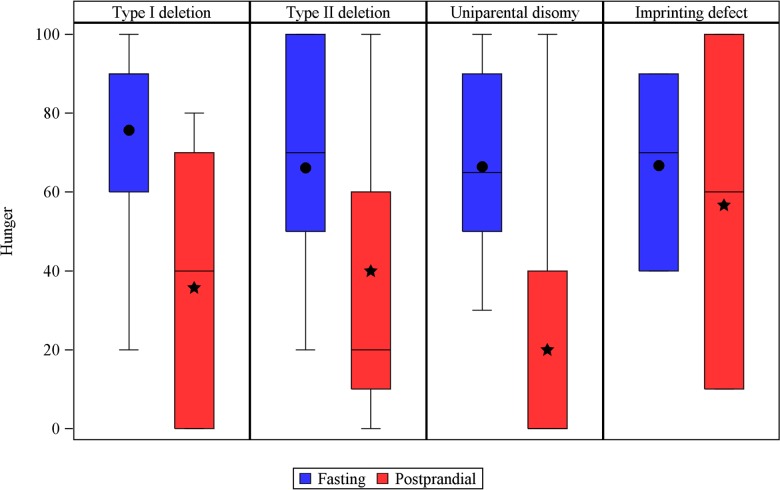
Hunger score at fasting (circles) versus 60’ after the meal (stars) in the different genetic subtypes of Prader-Willi syndrome subjects. P = 0.034 for uniparental disomy, nonsignificant for the rest. Hunger was quantified on a visual analogue scale from 0 to 100.

In the fasting state, hunger scores, like fasting BDNF levels, did not differ among genetic subtypes. However, after the meal, hunger scores differed with subtype (Fig 6). All subjects with imprinting defects (100%), most subjects with type II deletions (76.9%), and slightly more than half of those with type I deletions (57%) were still hungry 60 minutes after the meal. At this time point, the hunger score had decreased significantly only in the UPD subgroup, where only 28.6% of the subjects were still hungry (p = 0.05 between all groups). In UPD subjects, every increase of 50 units in baseline BDNF, decreased the odds of being hungry by 66% (OR: 0.34, 90%CI: 0.13–0.9), whereas in the other genetic subtypes, baseline BDNF was not associated with any significant effect on the odds of being hungry.

### Effects of previous treatment with growth hormone on BDNF, leptin, and hunger score

BDNF levels, leptin levels, and hunger scores did not differ between patients who had been treated with growth hormone before adulthood and those who had not (data not shown).

## Discussion

To our knowledge, this is the first study to analyze fasting and postprandial plasma BDNF concentrations in adults with PWS. A previous study in children with PWS found low peripheral BDNF (a reflection of central BDNF) that may contribute to the lack of satiety in this syndrome [33], and the present study extends these findings to adults. Moreover, we found that the postprandial peak in BDNF observed in lean controls at 60’ was absent in both PWS and obese subjects and could contribute to their hyperphagia. Interestingly, postprandial patterns of BDNF levels over time differed among genetic subtypes, and baseline BDNF levels seem to influence the odds of being hungry after a meal.

The regulation of energy homeostasis is complex, and many central and peripheral signals are involved in controlling hunger and satiety. BDNF is believed to act as a satiety signal downstream of leptin-melanocortin signaling [34]; however, no relationship between peripheral leptin and plasma/serum BDNF concentrations has been demonstrated [33,35]. Interestingly, in the present study the lowest BDNF/leptin quotient was seen in PWS subjects. Moreover, as was previously found in children [36], leptin levels in our adult subjects with PWS were higher than in lean controls, suggesting that the defect in energy homeostasis lies beyond leptin production in the adipocyte, as if there were a resistance to the action of leptin. This defect could affect transport across the blood-brain barrier or it could affect BDNF production downstream of leptin-melanocortin signaling.

Leptin and BDNF are both necessary and act reciprocally to induce satiety [23]. Leptin can stimulate the translation of long 3’UTR BDNF mRNA in neuronal dendrites through neuronal activity. BDNF derived from this type of transcription is then required for leptin-induced neuronal activity in several hypothalamic areas, where it probably regulates the formation, maintenance, and function of neuronal connections in several cortical and hippocampal areas. When BDNF signaling is compromised, neural circuits in these areas are dysfunctional, leading to low local BDNF levels and therefore low peripheral concentrations (as a reflection of central BDNF); consequently, adipocytes secrete more leptin, and as a result of leptin resistance, hunger persists, leading to obesity. The present results are consistent with these observations since adults with PWS had low fasting BDNF levels and no postprandial peak, independent of hunger score. Moreover, higher fasting and postprandial plasma leptin concentrations in PWS subjects and obese controls could reflect leptin resistance, suggesting that BDNF and leptin may contribute, at least in part, to lack of satiety.

On the other hand, necdin, a signaling protein encoded by a gene located at 15q11-q12 that is not expressed in PWS, modulates signals of BDNF by binding to its receptor. Both BDNF and its receptor are coexpressed in hypothalamic nuclei associated with satiety, and the lack of necdin promotes adipogenesis [37]. In the present study, the low peripheral BDNF levels in PWS and baseline BDNF’s ability to predict postprandial hunger also support the hypothesis of reduced central BDNF action leading to a lack of satiety and a predisposition to obesity. Interestingly, obese subjects' BDNF pattern was similar to that of lean controls but without a significant peak at 60’, making it intermediate between the patterns observed in PWS and lean subjects. Thus, although postprandial hunger was similar in obese and lean controls, the lack of BDNF peak might predispose obese subjects to adipogenesis. These findings evidence the complexity of the regulation of postprandial satiety circuits in obesity, whether syndromic or not.

The pattern of postprandial plasma BDNF levels differed among genetic subtypes of PWS. In the UPD subtype, there was a sustained rise in BDNF over time, and a smaller proportion of subjects were hungry after the meal. These findings agree with other authors’ observations that UPD subjects have better self-control regarding food and more brain activity in the dorsolateral prefrontal cortex and parahippocampal gyrus, regions involved in cognitive control over decision-making (38). The less favorable postprandial BDNF pattern in subjects with type I and II deletions or imprinting defects and the persistence of hunger after the meal in these subtypes also make sense in light of the greater activity reported in these subtypes in brain regions involved in the motivation to eat, such as prefrontal cortex and amygdala [38], as well as with their higher tendency to overeat and to steal food, observed especially in patients with deletions [4].

Whether growth hormone treatment decreases leptin levels is controversial [39,40]. We found no differences in plasma leptin or BDNF concentrations in patients that had been treated with growth hormone before adulthood and those who had not.

One limitation of this study is the small number of subjects in each subtype, so caution is warranted in interpreting our results until they can be corroborated in further studies with more patients. Furthermore, central BDNF would have been a more reliable marker than peripheral BDNF, but determining central BDNF would have been more technically complex. Moreover, as BDNF is stored peripherally in platelets, we corrected the results in function of platelet counts, and the results remained unchanged. Likewise, corrections for body fat distribution and HOMA_IR-index did not change the results.

In summary, our findings suggest that BDNF levels play a role in hyperphagia in subjects with PWS; low baseline BDNF levels and a lack of postprandial BDNF peak may be related to the persistence of hunger after a meal. Further studies with more patients are needed to corroborate the differences in postprandial BDNF patterns among the different genetic subtypes.

## References

[1] Cassidy SB, Schwartz S, Miller JL, Driscoll DJ. Prader-Willi syndrome. Genet Med. 2012;14:10–26. 10.1038/gim.0b013e31822bead0 22237428

[2] Holm VA, Cassidy SB, Butler MG, Hanchett JM, Greenswag LR, Whitman BY, et al Prader-Willi syndrome: consensus diagnostic criteria. Pediatrics. 1993; 91:398–402. 8424017 PMC6714046

[3] Burman P, Ritzen EM, Lindgre AC. Endocrine dysfunction in Prader-Willi syndrome: a review with special reference to GH. Endocr Rev. 2001; 22:787–799. 10.1210/edrv.22.6.0447 11739333

[4] Butler MG, Bittel DC, Kibiryeva N, Talebizadeh Z, Thompson T. Behavioral differences among subjects with Prader-Willi syndrome and type I or type II deletion and maternal disomy. Pediatrics. 2004; 113:565–573. 10.1542/peds.113.3.565 14993551 PMC6743499

[5] Giménez-Palop O, Giménez-Pérez G, Mauricio D, Potau N, Berlanga E, González-Clemente JM, et al A lesser postprandial suppression of plasma ghrelin in Prader-Willi syndrome is associated with low fasting and a blunted postprandial PYY response. Clin Endocrinol. 2007; 66:198–204. 10.1111/j.1365-2265.2006.02707.x 17223988

[6] Cummings DE, Clement K, Purnell JQ, Vaisse C, Foster KE, Frayo RS, et al Elevated plasma ghrelin levels in Prader-Willi syndrome. Nat Med. 2002; 8:643–644. 10.1038/nm0702-643 12091883

[7] Delparigi A, Tschöp M, Heiman Ml, Salbe AD, Vozarova B, Sell SM, et al High circulating ghrelin: a potential cause for hyperphagia and obesity in Prader-Willi Syndrome. J Clin Endocrinol Metab. 2002; 87:5461–5464. 10.1210/jc.2002-020871 12466337

[8] Haqq AM, Stadler DD, Rosenfeld RG, Pratt KL, Weigle DS, Frayo RS, et al Circulating ghrelin levels are suppressed by meals and octreotide therapy in children with Prader-Willi Syndrome. J Clin Endocrinol Metab. 2003; 88:3573–3576. 10.1210/jc.2003-030205 12915638

[9] Höybye C, Barkeling B, Espelund U, Petersson M, Thorén M. Peptides associated with hyperphagia in adults with Prader-Willi syndrome before and during GH treatment. Growth Horm Res. 2003; 13:322–327. 10.1016/s1096-6374(03)00077-7 14624765

[10] Zipf WB, O’Dorisio TM, Cataland S, Sotos J. Blunted pancreatic polypeptide responses in children with obesity of Prader-Willi syndrome. J Clin Endocrinol Metab. 1981; 52:1264–1266. 10.1210/jcem-52-6-1264 7014602

[11] Butler MG, Bittel CD, Talebizadeh Z. Plasma peptide YY and ghrelin levels in infants and children with Prader-Willi syndrome. J Pediatr Endocrinol Metab. 2004; 17:1177–1184. 10.1515/jpem.2004.17.9.1177 15506676 PMC5176014

[12] Bueno G, Moreno LA; Pineda I, Campos J, Ruibal JL, Juste MG, et al Serum leptin concentrations in children with Prader-Willi syndrome and non-syndromal obesity. J Pediatr Endocrinol Metab. 2000; 13:425–430. 10776997 10.1515/JPEM.2000.13.4.425

[13] Proto C, Romualdi D, Cento RM, Romano C, Campagna G, Lanzone A. Free and total leptin serum levels and soluble leptin receptors levels in two models of genetic obesity: the Prader-Willi and the Down syndromes. Metabolism. 2007; 56:1076–1080. 10.1016/j.metabol.2007.03.016 17618952

[14] Myers MG Jr, Leibel RL, Seeley RJ, Schwartz MW. Obesity and leptin resistance: distinguishing cause from effect. Trends Endocrinol Metab. 2010; 21:643–651. 10.1016/j.tem.2010.08.002 20846876 PMC2967652

[15] Hirsch HJ, Gross I, Pollak Y, Eldar-Geva T, Gross-Tsur V. Irisin and the metabolic phenotype of adults with Prader-Willi syndrome. Plos One. 2015; 10: e0136864 10.1371/journal.pone.0126864 ecollection 2015 26334732 PMC4559418

[16] Tan TM, Vanderpump M, Khoo B, Patterson M, Ghatei MA, Goldstone AP. Somatostatin infusion lowers plasma ghrelin without reducing appetite in adults with Prader-Willi syndrome. J Clin Endocrinol Metab. 2004; 89:4162–4165. 10.1210/jc.2004-0835 15292365

[17] Goldstone AP, Patterson M, Kalingag N, Ghatei MA, Brynes AE, Bloom SR, et al Fasting and post-prandial hyperghrelinemia in Prader-Willi syndrome is partially explained by hypoinsulinemia, and is not due to peptide YY_3-36_ deficiency or seen in hypothalamic obesity due to craneopharingioma. J Clin Endocrinol Metab. 2005; 90:2681–2690. 10.1210/jc.2003-032209 15687345

[18] Caixàs A, Giménez-Palop O, Giménez-Pérez G, Potau N, Berlanga E, González-Clemente JM, et al Postprandial adiponectin levels are unlikely to contribute to the pathogenesis of obesity in Prader-Willi syndrome. Horm Res. 2006; 65:39–45. 10.1159/000090513 16374018

[19] Rigamonti AE, Bini S, Grugni G, Agosti F, De Col A, Mallone M, et al Unexpectedly increased anorexigenic postprandial responses of PYY and GLP-1 to fast ice cream consumption in adult patients with Prader-Willi syndrome. Clin Endocrinol. 2014; 81:542–550. 10.1111/cen.12395 24372155

[20] Caixàs A, Giménez-Palop O, Broch M, Vilardell C, Megía A, Simón I, et al Adult subjects with Prader-Willi syndrome show more low-grade systemic inflammation than matched obese subjects. J Endocrinol Invest. 2008; 31: 169–175. 10.1007/BF03345585 18362510

[21] Henderson CE, Bloch-Gallego E, Camu W, Gouin A, Mettling C. Neurotrophic factors in development and plasticity of spinal neurons. Restor Neurol Neurosci. 1993; 5:15–28. 10.3233/RNN-1993-5105 21551684

[22] Fujimura H, Altar C, Chen R, Nakamura T, Nakahashi T, Kambayashi J, et al Brain-derived neurotrophic factor is stored in human platelets and released by agonist stimulation. Thromb Haemost. 2002; 87:728–734. 12008958

[23] Liao GY, An JJ, Gharami K, Waterhouse EG, Vanevski F, Jones KR, et al Dendritically targeted Bdnf mRNA is essential for energy balance and response to leptin. Nat Med. 2012;18:564–571 10.1038/nm.2687 22426422 PMC3327556

[24] Thoenen H. Neurotrophins and neuronal plasticity. Science. 1995; 270:593–598. 10.1126/science.270.5236.593 7570017

[25] Bulló M, Peeraully M, Trayhurn P, Folch J, Salas-Salvadó J. Circulating nerve growth factor levels in relation to obesity and the metabolic syndrome in women. Eur J Endocrinol. 2007; 157:303–310. 10.1530/EJE-06-0716 17766712

[26] Lapchak PA, Hefti F. BDNF and NGF treatment in lesioned rats: effects on cholinergic function and weight gain. Neuroreport. 1992; 3:405–408. 10.1097/00001756-199205000-00007 1633277

[27] Han JC, Liu QR, Jones M, Levinn RL, Menzie CM, Jefferson-George KS, et al Brain-derived neurotrophic factor and obesity in the WAGR syndrome. N Engl J Med. 2008; 359:918–927. 10.1056/NEJMoa0801119 18753648 PMC2553704

[28] Gray J, Yeo GS, Cox JJ, Morton J, Adlam AL, Keogh JM, et al Hyperphagia, severe obesity, impaired cognitive function, and hyperactivity associated with functional loss of one copy of the brain-derived neurotrophic factor (BDNF) gene. Diabetes. 2006; 55:3366–3371. 10.2337/db06-0550 17130481 PMC2413291

[29] Hall D, Dhilla A, Charalambous A, Gogos JA, Karayiorgou M. Sequence variants of the brain-derived neurotrophic factor (BDNF) gener are strongly associated with obsessive-compulsive disorder. Am J Hum Genet. 2003; 73:370–376. 10.1086/377003 12836135 PMC1180373

[30] Geller B, Badner JA, Tillman R, Christian SL, Bolhofner K, Cook EH Jr. Linkage disequilibrium of the brain-derived neurotrophic factor Val66Met polymorphism in children with a prepubertal and early adolescent bipolar disorder phenotype. Am J Psychiatry. 2004; 161:1698–1700. 10.1176/appi.ajp.161.9.1698 15337662

[31] Ribasés M, Gratacòs M, Fernandez-Aranda F, Bellodi L, Boni C, Anderluh M, et al Association of BDNF with anorexia, bulimia and age of onset of weight loss in six European populations. Hum Mol Genet. 2004; 13:1205–1212. 10.1093/hmg/ddh137 15115760

[32] Corripio R, González-Clemente JM, Pérez-Sánchez J, Näf S, Gallart L, Vendrell J, et al Plasma brain-derived neurotrophic factor in prepubertal obese children: results from a 2-year lifestyle intervention programme. Clin Endocrinol. 2012; 77:715–720. 10.1111/j.1365-2265.2012.04431.x 22563866

[33] Han JC, Muehlbauer MJ, Cui HN, Newgard CB, Haqq AM. Lower brain-derived neurotrophic factor in patients with Prader-Willi syndrome compared to obese and lean control subjects. J Clin Endocrinol Metab. 2010; 95:3532–3536. 10.1210/jc.2010-0127 20427492 PMC2928902

[34] Xu B, Goulding EH, Zang K, Cepoi D, Cone RD, Jones KR, et al Brain-derived neurotrophic factor regulates energy balance downstream of melanocortin-4 receptor. Nat Neurosci. 2003; 6:736–742. 10.1038/nn1073 12796784 PMC2710100

[35] Haqq AM, Muehlbauer M, Svetkey LP, Newgard CB, Purnell JQ, Grambow SC, et al Altered distribution of adiponectin isoforms in children with Prader-Willi syndrome (PWS): association with insulin sensitivity and circulating satiety peptide hormones. Clin Endocrinol. 2007;67:944–951. 10.1111/j.1365-2265.2007.02991.x 17666087 PMC2605973

[36] Goldstone AP, Holland AJ, Butler JV, Whittington JE. Appetite hormones and the transition to hyperphagia in children with Prader-Willi syndrome. Int J Obes (Lond). 2012; 36:1564–1570. 10.1038/ijo.2011.274 22270375

[37] Bush JR, Wevrick R. Loss of the Prader-Willi obesity syndrome protein necdin promotes adipogenesis. Gene. 2012; 497:45–51. 10.1016/j.gene.2012.01.027 22305984

[38] Holsen LM, Zarcone JR, Chambers R, Butler MG, Bittel DC, Brooks WM, et al Genetic subtype differences in neural circuitry of food motivation in Prader-Willi syndrome. Int J Obes (Lond). 2009; 33:273–283. 10.1038/ijo.2008.255 19048015 PMC2643328

[39] Irizarry KA, Bain J, Butler MG, Ilkayeva O, Muehlbauer M, Haqq AM, et al Metabolic profiling in Prader–Willi syndrome and nonsyndromic obesity: sex differences and the role of growth hormone. Clin Endocrinol. 2015; 83: 797–805. 10.1111/cen.12766 25736874 PMC4560678

[40] Höybye C. Endocrine and metabolic aspects of adult Prader-Willi syndrome with special emphasis on the effect of growth hormone treatment. Growth Horm IGF Res. 2004; 14:1–15. 10.1016/j.ghir.2003.09.003 14700552

